# Genetic Analysis of KRT9 Gene Revealed Previously Known Mutations and Genotype-Phenotype Correlations in Epidermolytic Palmoplantar Keratoderma

**DOI:** 10.3389/fgene.2018.00645

**Published:** 2019-01-07

**Authors:** Yuwei Li, Lili Tang, Yang Han, Liyun Zheng, Qi Zhen, Sen Yang, Min Gao

**Affiliations:** ^1^Institute of Dermatology and Department of Dermatology of First Affiliated Hospital, Hefei, China; ^2^Key Laboratory of Dermatology, Ministry of Education, Anhui Medical University, Hefei, China

**Keywords:** epidermolytic palmoplantar keratoderma, gene mutation, hot spot, KRT9 gene, knuckle pads

## Abstract

Epidermolytic palmoplantar keratoderma (EPPK, OMIM 144200) is an autosomal dominant inherited disease, clinically characterized by diffuse yellowish thickening of the skin on the palms and soles, usually with erythematous borders developing during the first weeks or months after birth. Pathogenesis of EPPK is determined by mutations in the keratin gene (KRT9). Thirty three mutations in the KRT9 gene from 100 EPPK families have been identified. Among these, 23 mutations are located in the 1A region (a mutation hot spot region), 7 are located in the 2B region, and the remaining 3 are synonymous mutations. In this study, three heterozygous mutations (p.N161S, p.R163W, and p.R163Q), located in regions of the gene encoding the conserved central a-helix rod domain, were detected in the KRT9 gene of the three large Chinese families. This study confirms that codon 163 (48 of 100 cases) is a hot spot mutation site for KRT9. Additional findings identified p.N161S (4%) and p.R163W (4%) as potential hot spot mutations for EPPK associated with knuckle pads, and p.R163Q (15 of 100 cases) as the hot spot mutation of EPPK not occurring in combination with knuckle pads. In conjunction with future studies, this research may help lay the foundation for genetics counseling, prenatal diagnosis and clinical treatment of EPPK.

## Background

Epidermolytic palmoplantar keratoderma (EPPK, OMIM 144200), also known as Vorner's palmoplantar keratosis, is an autosomal dominant inherited disease characterized by diffuse, yellow thickening of the palm and sole with an erythematous margin. It was first described in 1901 by Vorner. The incidence rate is approximately 2.2 to 4.4 per 100 000 live newborns (Bonifas et al., 1994; Covello et al., 1998; Smith, 2003; Lopez-Valdez et al., 2013; Liu et al., 2014). Some patients may have hyperhidrosis, knuckle pads, camptodactyly and digital mutilation (Lu et al., 2003; Du et al., 2011; Umegaki et al., 2011). Female patients may have an increased risk for ovarian cancer or breast cancer (Hamada et al., 2013).

Keratin 9 is composed of the functional head domain, the functional α-helical domain and the functional tail domain, and is expressed only in the suprabasal layers of the palmoplantar epidermis (Uitto et al., 2007). To date, domestic and foreign scholars have found 33 KRT9 gene mutations in 100 EPPK families, of which 15 cases are associated with knuckle pads. There is no report making a detailed summary and analysis. In this study, cases of EPPK were analyzed to look for genotype-phenotype correlations by searching the database and consulting the literatures, providing a theoretical basis for the prenatal diagnosis of, genetic counseling for, and clinical treatment of EPPK.

## Compliance With Ethical Standards

All procedures performed in studies involving human participants were in accordance with the ethical standards of the institutional and/or national research committee and with the 1964 Helsinki declaration and its later amendments or comparable ethical standards. Informed written consents were obtained from all individual participants or their legal representatives (parents) included in the study. The study was approved without restrictions by the Medical Ethics Committee of the First Affiliated Hospital of Anhui Medical University. The probands and their family members provided written informed consent for the publication of this case report.

## Case Presentation

**Figure 1:**
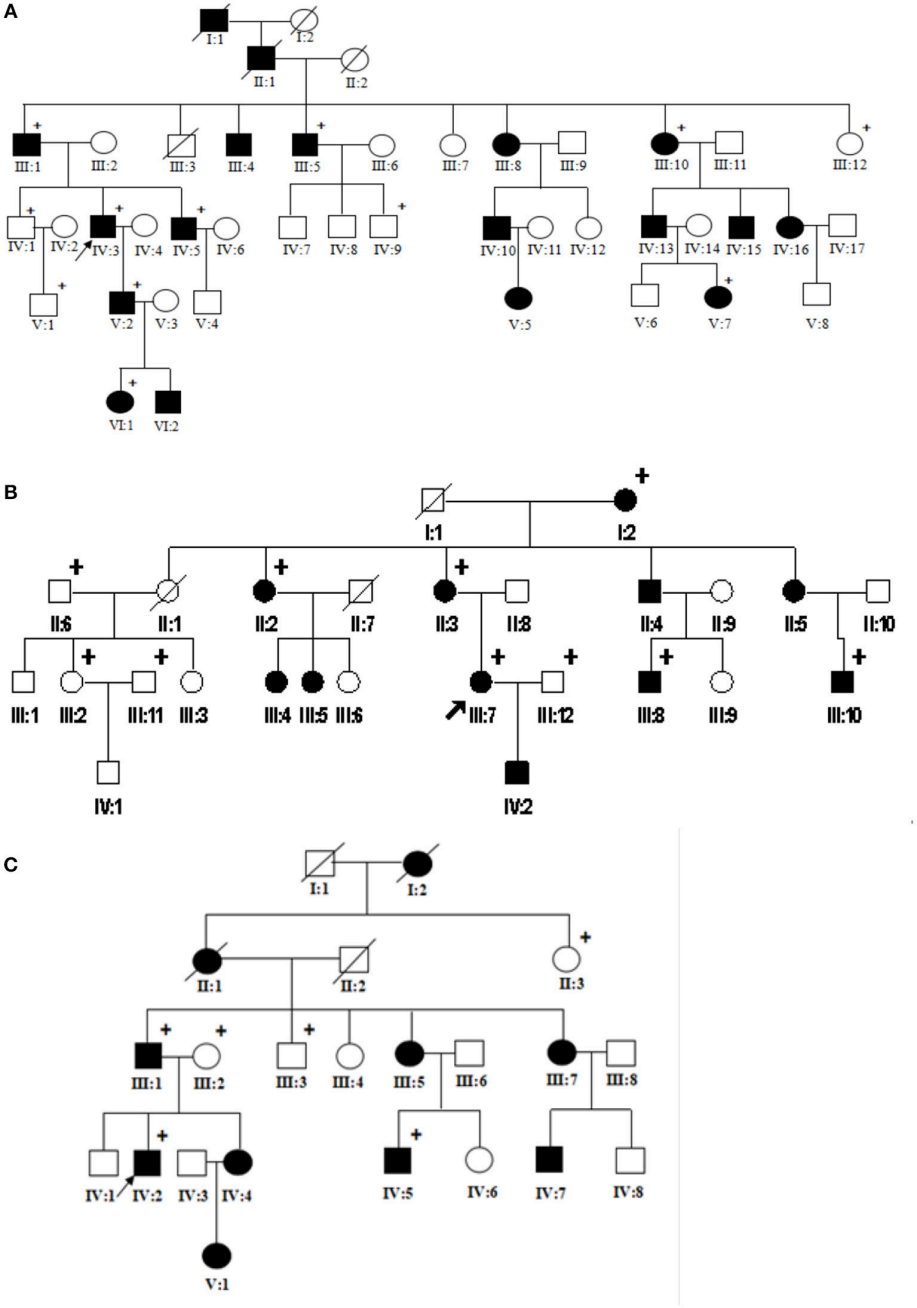
Pedigrees of EPPK families 1 **(A)**, 2 **(B)**, and 3 **(C)**. Arrows show the probands.

**Figure 2:**
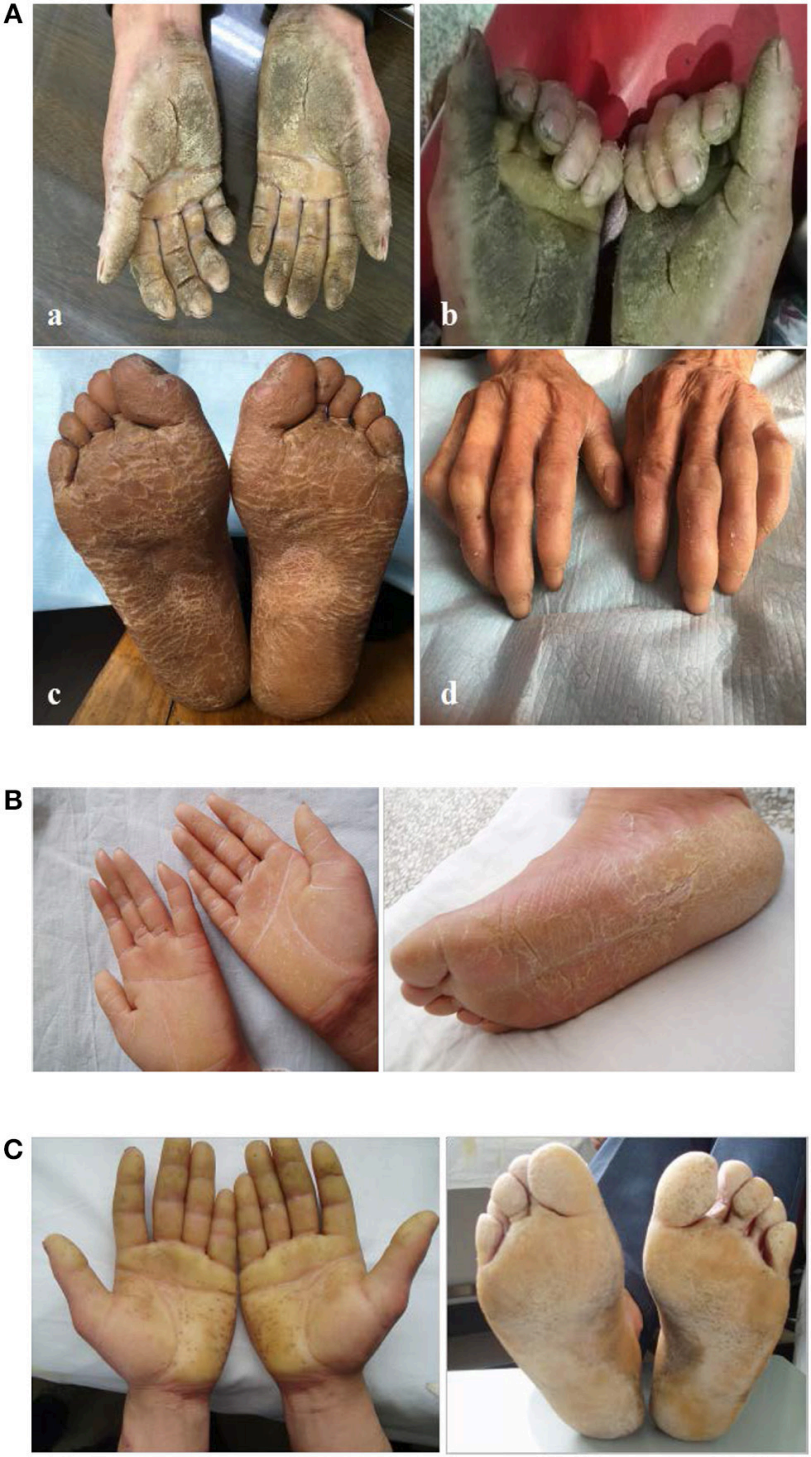
Clinical representations of the proband. **(A)** Knuckle pads on the dorsal aspect of the proband's hands. **(B)** Diffuse hyperkeratosis of the proband's palms and soles. **(C)** Well-demarcated erythematous border of the proband's.

Three unrelated Chinese EPPK pedigrees from Shandong and Anhui Province were investigated. All patients exhibited typical EPPK features. There were no close relatives bettwen these families. Family 1 was a six generational EPPK pedigree with 17 affected members, including 11 males and 6 females. The minimum age of onset is 1 year of age. The proband was a 42-year-old man who presented with diffuse thickening and hyperkeratosis on palms with the nails being normal, and combined knuckle pads, hyperhidrosis and camptodactyly (Figures 1A, 2A). There is no evidence that the proband was associated with other diseases. Family 2 was a 4 generational EPPK pedigree with 11 affected members, and the 20-year-old female proband presented with diffuse thickening and hyperkeratosis on palms and soles (Figures 1B, 2B). Family 3 was a 5 generational EPPK pedigree with 10 affected members, and the proband was a 42-year-old man who demonstrated hyperkeratosis of both palmar and plantar skin within 1 year of birth (Figures 1C, 2C).

## Laboratory Investigations

### Genetic Testing and Confirmation of Mutation

Peripheral blood samples were collected from members of the three families and 100 healthy unrelated Chinese individuals. Genomic DNA was extracted using a Flexi Gene DNA Kit (250). The primers were amplified by polymerase chain reaction (PCR) and PCR products were directly sequenced by an ABI3730 DNA Sequencer (ABI, USA). The sequence was analyzed by Chromas 2.0 software.

### Identification of Three Distinct Mutations in the KRT9 Gene of Three Large Chinese Families

**Figure 3:**
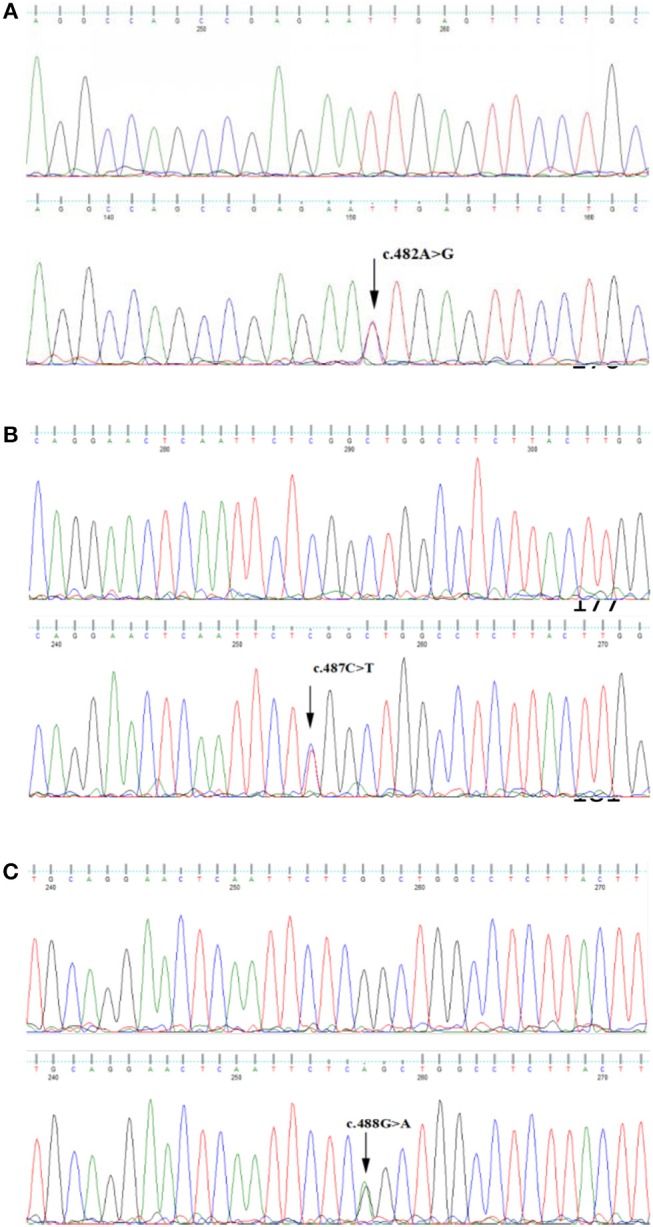
**(A)** Heterozygous variants c.482A>G identified in Family PPK-1. **(B)** Heterozygous variants c.487C>T identified in Family PPK-2. **(C)** Heterozygous variants c.488G>A identified in Family PPK-3.

Patients of family 1 had a heterozygous mutation c.482A>G (P.Asn161His) in KRT9 gene (Figure 3A). Patients of family 2 had a heterozygous mutation c.487C>T (p.Arg163Trp) in KRT9 gene (Figure 3B). Patients of family 3 had a heterozygous mutation c.488G>A (p.Arg163Gln) in KRT9 gene (Figure 3C).These mutations were not found in normal members of three families and in 100 healthy controls.

### Genetic Characteristics of the Mutation in the KRT9 Gene

This study searched the human intermediate filament database (http://www.interfil.org/index.php), PubMed (https://www.ncbi.nlm.nih.gov/pubmed), China National Knowledge Internet (http://www.cnki.net/), and a large portion of literature and found that 33 KRT9 gene mutations were reported in 100 EPPK families by domestic and foreign scholars (Table 1). Disease causing mutations are as follows: of which 23 are located in the 1A region (hot spot mutation region), 7 in the 2B region, and the remaining 3 are synonymous mutations. Missense mutations at amino acid 163 (48% of all mutations) is indeed a hot spot mutation site for KRT9. We also found that the mutations of EPPK associated with knuckle pads (15% of 100 cases) are p.N161S (4%), p.R163W (4%), p.L168S (3%), p.M157T (1%), p.L160F (1%), p.C406R (1%), and p.L458p (1%). Since these mutations are the most prevalent we can suggest that p.N161S and p.R163W are potential hot spot mutations of EPPK associated with knuckle pads. The hot spot mutation of EPPK not associated with knuckle pads is p.R163Q (15 of 100 cases).

**Table 1:** Mutations analysis in *KRT9* gene of EPPK.

**No**	**Nucleotide change**	**Amino acid changes**	**Domain**	**Clinical symptoms**	**Number of reported cases**	**References**
1	c.31T>G; 31_516del	p.Leu11Val; Leu11_Gln172 del	head, 1A	EPPK	1	Fuchs-Telem et al., 2013
2	c.469A>G	p.Met157Val	1A	EPPK	2	Hennies et al., 1994
					2	Covello et al., 1998
					1	Rugg et al., 2002
3	c.470T>C	p.Met157Thr	1A	EPPK without knuckle pads	1	Covello et al., 1998
					1	Shimomura et al., 2010
				EPPK with knuckle pads	1	Chen et al., 2009
4	c.470T>G	p.Met157Arg	1A	EPPK	1	Shimazu et al., 2006
					1	Zhao et al., 2008
					1	Liang et al., 2014
5	c.470T>A	p.Met157Lys	1A	EPPK	1	Shimomura et al., 2010
6	c.478C>G	p.Leu160Val	1A	EPPK	1	(Endo and Hatamochi, 1997)
7	c.478C>T	p.Leu160Phe	1A	EPPK with knuckle pads	1	Lu et al., 2003
8	c.481A>T	p.Asn161Tyr	1A	EPPK	1	Torchard et al., 1994
9	c.481A>C	p.Asn161His	1A	EPPK	1	Lee et al., 2003
					1	Lin et al., 2004
10	c.482A>G	p.Asn161Ser	1A	EPPK without knuckle pads	1	Bonifas et al., 1994
					1	Amichai et al., 2002
					1	Lee et al., 2003
					1	Liu et al., 2014
					1	Mao et al., 2018
				EPPK with knuckle pads	1	Tsunemi et al., 2002
					1	Zhang et al., 2004
					1	Hamada et al., 2005
					1	Yin et al., 2007
11	c.482A>T	p.Asn161Ile	1A	EPPK	1	Kuster et al., 2002
					1	Csikós et al., 2003
12	c.483T>A	p.Asn161Lys	1A	EPPK	1	Reis et al., 1994
13	c.484C>T	p.Pro162Ser	1A	EPPK	1	Li et al., 2008
14	c.484T>C	p.Ser162Pro	1A	EPPK	1	Zeng et al., 2017
15	c.487C>T	p.Arg163Trp	1A	EPPK without knuckle pads	3	Reis et al., 1994
					1	Bonifas et al., 1994
					2	Navsaria et al., 1995
					1	Rothnagel et al., 1995
					1	Yang et al., 1998
					1	Mayuzumi et al., 1999
					1	Morgan et al., 1999
					1	Warmuth et al., 2000
					1	Rugg et al., 2002
					1	Yang et al., 2003
					3	Lee et al., 2003
					3	Terrinoni et al., 2004
					1	Funakushi et al., 2009
					1	Umegaki et al., 2011
					2	Liu et al., 2012
					1	Guo et al., 2014
					2	Ke et al., 2014
					1	Wang et al., 2016
				EPPK with knuckle pads	1	Mao et al., 2018
					1	Chiu et al., 2007
					1	Codispoti et al., 2009
					1	Lopez-Valdez et al., 2013
16	c.488G>A	p.Arg163Gln	1A	EPPK	1	Reis et al., 1994
					1	Kobayashi et al., 1996
					1	Yang et al., 1998
					1	Covello et al., 1998
					1	Szalai et al., 1999
					1	Rugg et al., 2002
					1	Wennerstrand et al., 2003
					1	Yang et al., 2003
					1	Sun et al., 2005
					2	Shimomura et al., 2010
					1	Li et al., 2012
					1	Ke et al., 2014
					1	Zhang et al., 2016
					1	Mao et al., 2018
17	c.488G>C	p.Arg163Pro	1A	EPPK	1	Kon et al., 2006
18	c.491T>C	p.Leu164Pro	1A	EPPK	1	Mao et al., 2018
19	c.500_500delAinsGGCT	p.Tyr167delinsTrpLeu	1A	EPPK	1	He et al., 2004
					3	Zhang et al., 2005
20	c.503T>C	p.Leu168Ser	1A	EPPK with knuckle pads	1	Rothnagel et al., 1995
					1	Yin et al., 2007
					1	Li et al., 2009
21	c.508A>T	p.Lys170X	1A	EPPK	1	Szalai et al., 1999
22	c.511G>A	p.Val171Met	1A	EPPK	1	Rugg et al., 2002
23	c.515A>C	p.Gln172Pro	1A	EPPK	1	Hennies et al., 1994
24	c.1216T>C	p.Cys406Arg	2B	EPPK with knuckle pads	1	Wang et al., 2010
25	c.1282C>T	p.Gln428X	2B	EPPK	1	Umegaki et al., 2011
26	c.1360T>C	p.Tyr454His	2B	EPPK	1	Shimomura et al., 2010
27	c.1362_1363insCAC	p.Tyr454_His 455insHis	2B	EPPK	1	Coleman et al., 1999
28	c.1369C>T	p. Leu457Phe	2B	EPPK	1	Xiao et al., 2018
29	c.1372C>T	p.Leu458Phe	2B	EPPK	1	Kon et al., 2006
30	c.1373 T >C	p.L458P	2B	EPPK with knuckle pads	1	Du et al., 2011

*3 synonymous mutations are not listed*.

## Discussion

Epidermolytic palmar hyperkeratosis (EPPK) is rare in clinical settings with a prevalence of ~4.4/100,000. In mild cases, only the epidermis of the palmoplantar is rough, and severe horny thickening plaques appear in the palmoplantar region. It may even spread to the lateral edge of the palmoplantar skin or the hands and feet and may be accompanied by knuckle pads and finger-toe flexion deformities. The keratin mutations that have been found so far are concentrated in the 1A helix region and the 2B helix region, namely, the KRT9 gene mutation hot spot (Guo et al., 2014; Liang et al., 2014), especially the 1A region, which affects the formation of keratinous network structures leading to severe clinical manifestations.

Researchers at home and abroad have found that the 163rd amino acid is the hotspot mutation region of the KRT9 gene (48 of 100 cases) (Rugg et al., 2002). EPPK combined with knuckle pads maybe associated with mutations in many KRT9 genes, such as p.Met157Thr, p.Leu160Phe, p.Asn161Ser,p.Arg163Trp, p.Leu168Ser, p.Cys406Arg, and p.Leu458Pro (Escobar et al., 2007; Codispoti et al., 2009; Li et al., 2009; Wang et al., 2010; Du et al., 2011; Mao et al., 2018; Xiao et al., 2018). In this study, we studied three large Chinese families with EPPK and found 3 heterozygous gene mutations of KRT9: c.482A>G (p.Asn161Ser), c.487C>T (p.Arg163Trp) and c.488G>A (p.Arg163Gln).These mutations were not found in normal members of three families and in 100 healthy controls, indicating that the mutations detected in the families were the pathogenic mutations. The mutation p.Asn161Ser has been reported several times in relation to the typical hyperkeratotic manifestations of palmoplantar skin in patients with EPPK, but it has not been associated with other clinical phenotypes. In 2005, Japanese scholars reported a case of EPPK in a 13-year-old patient with knuckle pads, and the genetic test results were found to be associated with the mutation p.Asn161Ser. In this study, the pathogenic mutation of family 1 was p.Asn161Ser, and all patients in the family showed knuckle pads, consistent with previous reports. This study found that 33 KRT9 gene mutations in 100 EPPK families have been reported by domestic and foreign scholars, and the mutations of EPPK associated with knuckle pads (15 of 100 cases) are p.N161S (4%), p.R163W (4%), p.L168S (3%), p.M157T (1%), p.L160F (1%), p.C406R (1%), and p.L458p (1%), suggesting p.N161S and p.R163W are potential hot spot mutations of EPPK associated with knuckle pads, and p.R163Q (15 of 100 cases) as the hot spot mutation of EPPK not occurring in combination with knuckle pads.Based on the above studies, this case reveals knuckle pads may also be one of the less common clinical phenotypes of EPPK, and we think this study should help lay the foundation for genetics counseling, prenatal diagnosis and clinic treatment of EPPK.

## Author Contributions

All authors listed have made a substantial, direct and intellectual contribution to the work, and approved it for publication.

### Conflict of Interest Statement

The authors declare that the research was conducted in the absence of any commercial or financial relationships that could be construed as a potential conflict of interest.
